# Digestibility Is Similar between Commercial Diets That Provide Ingredients with Different Perceived Glycemic Responses and the Inaccuracy of Using the Modified Atwater Calculation to Calculate Metabolizable Energy

**DOI:** 10.3390/vetsci4040054

**Published:** 2017-11-08

**Authors:** Natalie J. Asaro, Marcial A. Guevara, Kimberley Berendt, Ruurd Zijlstra, Anna K. Shoveller

**Affiliations:** 1 Department of Animal Biosciences, University of Guelph, 50 Stone Road East, Guelph, ON N1G 2W1, Canada; nasaro@uoguelph.ca; 2 The Iams Company, MARS Pet Care, 6571 OH-503 North, Lewisburg, OH 45338, USA; marcial.guevara@effem.com; 3 Department of Agricultural, Food and Nutritional Science, University of Alberta, 116 St & 85 Ave, Edmonton, AB T6G 2R3, Canada; berendt@ualberta.ca (K.B.); zijlstra@ualberta.ca (R.Z.)

**Keywords:** feline, carbohydrate, digestibility, metabolizable energy

## Abstract

Dietary starch is required for a dry, extruded kibble; the most common diet type for domesticated felines in North America. However, the amount and source of dietary starch may affect digestibility and metabolism of other macronutrients. The objectives of this study were to evaluate the effects of 3 commercial cat diets on in vivo and in vitro energy and macronutrient digestibility, and to analyze the accuracy of the modified Atwater equation. Dietary treatments differed in their perceived glycemic response (PGR) based on ingredient composition and carbohydrate content (34.1, 29.5, and 23.6% nitrogen-free extract for High, Medium, and LowPGR, respectively). A replicated 3 × 3 Latin square design was used, with 3 diets and 3 periods. In vivo apparent protein, fat, and organic matter digestibility differed among diets, while apparent dry matter digestibility did not. Cats were able to efficiently digest and absorb macronutrients from all diets. Furthermore, the modified Atwater equation underestimated measured metabolizable energy by approximately 12%. Thus, the modified Atwater equation does not accurately determine the metabolizable energy of high quality feline diets. Further research should focus on understanding carbohydrate metabolism in cats, and establishing an equation that accurately predicts the metabolizable energy of feline diets.

## 1. Introduction

A consumer-driven trend towards pet foods that are organic and utilize novel ingredients is currently growing in the pet food industry. Pet food companies commonly advertise their product using terms such as “grain-free”, which is often combined with low carbohydrate claims. However, a grain-free diet might not be as beneficial as advertised when compared to a commercial diet that includes grains as a carbohydrate source. Overall, the ability of cats to metabolize carbohydrates has been poorly defined, and research regarding an optimum inclusion level and type of carbohydrate for the domestic cat is lacking. Although current standards indicate that the domestic cat is an obligatory carnivorous species [1], felines have displayed the physiological capability to successfully metabolize multiple carbohydrate sources, and that high carbohydrate inclusion levels (35%) do not impair macronutrient digestibility [2]. Cats also exhibit a metabolic ability to alter macronutrient oxidation based on variable intake of carbohydrates and fats [3].

There are many benefits of including different forms of carbohydrates in pet food formulations. Starch is required to maintain shape and texture of dry, extruded kibble, while the process of extrusion, drying, and enrobing is the most cost-effective way to produce a stable, low moisture product that is resistant to microbial growth [4]. Extrusion at high temperatures also increases the digestibility of carbohydrates and starches in particular [5]. Consumers benefit from feeding kibble in terms of affordability, convenience, and assurance that their pets’ food will remain microbially safe for an extended period of time. Furthermore, cats fed diets high in carbohydrates are at a lower risk of adiposity and correlated negative metabolic outcomes compared to cats fed diets high in fat [3]. Though controversy exists regarding the impact of carbohydrates in feline diets, there is limited research investigating the mechanisms of carbohydrate digestion and metabolism in cats.

The energy density of feline diets is commonly expressed as metabolizable energy (ME) [6]. Routine ME measurements are not practical or financially feasible [7]; therefore, generally accepted Atwater equations are used to predict ME values and develop feeding guidelines. These equations assign coefficients for the 3 macronutrients: protein, carbohydrate (measured as N-free extract (NFE)), and fat, and exist as traditional [8] and modified [9]:

Traditional  ME (kcal/kg) = [4 × CP (%) + 4 × NFE (%) + 9 × crude fat (%)] × 10(1)

Modified  ME (kcal/kg) = [3.5 × CP (%) + 3.5 × NFE (%) + 8.5 × crude fat (%)] × 10(2)

Because the traditional Atwater equation has been found to overestimate ME [10], a modified Atwater equation was developed and is recommended to estimate diet ME for dogs and cats [9]. However, neither Atwater equation accurately predicts the ME value of pet foods, because the coefficients are unreliable [11]. Because of the inaccuracy, the National Research Council (NRC) has suggested a more accurate method that accounts for crude fiber and digestibility of energy while calculating the ME value of prepared cat foods [11]:

(3) $$\begin{array}{ll} \mathrm{Step}\;1 & \mathrm{GE}\;\left(\mathrm{kcal}\right)=\left(5.7\times g\;\mathrm{protein}\right)+\left(9.4\times g\;\mathrm{fat}\right)+\left(4.1\times \left(g\;\mathrm{NFE}+g\;\mathrm{crude}\;\mathrm{fiber}\right)\right)\\ \mathrm{Step}\;2 & \mathrm{Percentage}\;\mathrm{energy}\;\mathrm{digestibility}=87.9-\left(0.88\times \mathrm{percentage}\;\mathrm{crude}\;\mathrm{fiber}\;\mathrm{in}\;\mathrm{dry}\;\mathrm{matter}\right)\\ \mathrm{Step}\;3 & \mathrm{DE}\;\left(\mathrm{kcal}/g\right)=\left(\mathrm{GE}\times \mathrm{percentage}\;\mathrm{energy}\;\mathrm{digestibility}/100\right)\\ \mathrm{Step}\;4 & \mathrm{ME}\;\left(\mathrm{kcal}/g\right)=\mathrm{DE}-\left(0.77\times g\;\mathrm{protein}\right) \end{array}$$

The NRC has also proposed an alternate equation to estimate percentage energy digestibility using total dietary fiber (TDF), rather than crude fiber [11]:

Energy digestibility (%) = 95.6 − (0.89 × total dietary fiber (%) in dry matter) (4)

However, past labeling practices of pet food do not require total dietary fiber to be reported and thus crude fiber is often used to approximate energy digestibility. Having accurate estimates of the energy density of food is important to provide practitioners and pet owners with accurate feeding guidelines.

The objectives of this study were to: (1) Measure the ME value of 3 commercial diets differing in NFE content; (2) compare measured to predicted ME values using the Atwater and modified Atwater equations; (3) use digestibility values (both in vitro and in vivo) to determine if cats can efficiently metabolize diets differing in hypothetical glycemic responses. We hypothesized that (1) because the Atwater equations do not account for fiber or energy digestibility, measured and predicted ME values would differ within diet; (2) due to the high quality of ingredients included in current commercial diets, along with adequate cooking/processing and high temperatures involved in extrusion, no diet will be significantly more digestible than another, with all being highly digestible.

## 2. Materials and Methods

All procedures were reviewed and approved by Procter and Gamble Pet Care’s Institutional Animal Care and Use Committee and were in accordance with the United States Department of Agriculture (USDA) and the Association for Assessment and Accreditation of Laboratory Animal Care (AAALAC) guidelines. The Animal Utilization Protocol was 013-9127 (dated 17 March 2013).

### 2.1. Animals

Twelve cats (6 neutered males, 6 spayed females) of similar age (4.9 ± 1.2 year) and weight (4.4 ± 0.8 kg) were used for the present study. Cats were previously acclimated to housing facilities and metabolic cages. Cats received physical veterinary exams to ensure health prior to and during the study.

### 2.2. Experimental Diets and Design

Since the glycemic response to each diet was not measured, the term perceived glycemic response (PGR) was used to characterize the diets in the present study. The PGR refers to the expected glycemic response that would hypothetically result from consumption of each diet. This estimate was determined by looking at the known glycemic indexes of the main carbohydrate sources included in each diet. Diets were selected for containing ingredients that are predicted to elicit high, medium, and low glycemic responses, and named based on their respected PGR. Three diets were studied (Table 1): HighPGR: Purina ONE Chicken and Rice (Nestlé, St. Louis, MO, USA), MediumPGR: Iams Kitten Proactive Health (Procter & Gamble, Cincinatti, OH, USA), and LowPGR: Innova Dry Adult Cat Food (Procter & Gamble, Cincinatti, OH, USA). Purina ONE Chicken and Rice was chosen as the HighPGR diet due to its high inclusion of Brewer’s rice, a high glycemic index (GI) grain [2]. Iams Kitten Proactive health was comprised of ingredients including corn meal and sorghum, that both elicit a lower glycemic response than ingredients such as Brewer’s rice [12]. Lastly, Innova Dry Adult Cat Food was predicted to have the lowest PGR because of the use of barley, a low GI carbohydrate [13], and its high inclusion of protein.

The experimental design was a replicated 3 × 3 Latin square, with 3 diets and 3 periods, with each cat randomly receiving every diet and all diets equally represented within each period. Each period lasted 10 days, with 5 days of acclimation to diet immediately followed by 5 days of collections. Cats were fasted overnight and weighed prior to feeding the morning of day 1 and 6. Cats were fed to maintain body weight (BW) based on historical feeding and body weight records. Diet allowance was determined based on the calculated energy density (modified Atwater calculation) [9] of each diet and maintenance energy requirements for each cat. The amount fed averaged 44 ± 8 g/day (SEM) and ranged from 32 to 55 g/day. To encourage consumption of food, cats were fed once at 07:00 daily to 95% of their maintenance requirements. Food refusals were collected and weighed at 13:00. Cats had ad libitum access to fresh water.

During the acclimation (days 1 to 5), cats were housed in a one-room free-living group environment (13.94 m^2^) in an environmentally controlled facility (22 °C; 50–60% relative humidity) with a 12 h light:12 h dark cycle and natural light through windows. Cats were placed in individual metabolism cages (0.61 m length × 0.61 m width × 0.62 m height; Suburban Surgical Company, Wheeling, IL, USA) for feeding each day from 06:30 until 13:00, and were then moved back into their free-living environment.

During the collection periods (days 6 to 10), cats were housed in individual stainless-steel metabolism cages. Two labeled urine collection bottles per cat fitted with screened funnels and containing 10 mL HCl as preservative were placed under each cage. Feces and urine collections started at 08:00 on day 6. Feces were collected, weighed, and scored using a scale of 0–5 with 0 as no stool, 1 as watery liquid, and 5 as extremely dry (The Iams Company standard operating procedure for collections and scoring). Feces were frozen in bags for each cat at −16 °C until analysis. Urine bottles were emptied daily into bottles for each cat and refrigerated at 3 °C until analyses. Clinical observations were recorded, but none were noted throughout the study.

### 2.3. Chemical Analyses and Calculations

Urine composites were mixed thoroughly and two 50 mL urine sub-samples were prepared for subsequent analyses. An aliquot of each cat’s fecal composite was freeze-dried. Diet and freeze-dried feces were ground to fine particle matter using a hand grinder and analyzed. Proximate analyses were completed in triplicate for each of the 3 experimental diets and fecal samples using the Association of Official Analytical Chemists (AOAC) procedures [14]. Crude fat was analyzed following acid hydrolysis (954.02), and dry matter (DM) was determined by vacuum drying at 100 °C for 24 h (934.01). Nitrogen was determined by oxidation using a crude protein/nitrogen (CP/N) analyzer (990.03; Leco Corp., St Joseph, MI, USA) and crude protein (CP) was calculated. Crude fiber (CF) was analyzed through a ceramic fiber filtration method (962.09). Starch was analyzed by the glucoamylase method (979.10), and total carbohydrate content was approximated as the value for N-free extract (NFE) [7], calculated as:

NFE (%) = 100 − protein (%) − fat (%) − fiber (%) − ash (%) − moisture (%)(5)

Ash was measured after exposure at 550 °C for 4 h (942.05), P through spectrophotometry (964.06), and Ca by atomic absorption spectrometry with electrothermal furnace (968.08). Gross energy was determined by bomb calorimetry (C-2000; IKA Staufen, Germany). The digestible energy (DE) of each diet was calculated by subtracting energy lost in feces from the determined gross energy (GE) intake. The ME value of each diet was measured by subtracting energy in feces and urine from GE intake.

### 2.4. In Vitro Energy Digestibility

The 3-step in vitro energy digestibility technique described by Huang et al. [15] was used to quantify digestible energy. In vitro DM digestibility was calculated by deducting the residue DM from the sample DM followed by division by the sample DM. Organic matter (OM) digestibility, protein digestibility, and fat digestibility were calculated using similar methods. The in vitro energy digestibility was calculated using the following formula [15]:

(6) $$\mathrm{In}\;\mathrm{Vitro}\;\mathrm{Energy}\;\mathrm{Digestibility}=\;\frac{\left(\mathrm{sample}\;\mathrm{DM}\;\times \;\mathrm{sample}\;\mathrm{GE}\right)\;-\;\left(\mathrm{residue}\;\mathrm{DM}\;\times \;\mathrm{residue}\;\mathrm{GE}\right)}{\left(\mathrm{sample}\;\mathrm{DM}\;\times \;\mathrm{sample}\;\mathrm{GE}\right)}$$

### 2.5. Statistical Analyses

Data were analyzed using the GLIMMIX procedure of SAS (version 9.3, SAS Inst., Cary, NC, USA) with cat as experimental unit, cat and period as random effects, and diet as fixed effect. Means were separated using the least significant difference. An alpha of 0.05 was used to declare statistical significance. Data were reported as least-squares means +/− SEM.

## 3. Results

When diets were analyzed, differences were detected in protein content, crude fat, and available lysine, with small differences in ash, acid detergent fiber (ADF) and neutral detergent fiber (NDF) (Table 1). The variation in nutrient levels were expected and occurred due to differences in ingredient composition of these diets.

Body weight was similar among groups at the beginning of the study (*p* > 0.05), and did not change throughout the study (*p* > 0.05). Cats fed the LowPGR diet excreted the most feces on an as-is basis, followed by cats fed the MediumPGR diet, and was the lowest for cats fed the HighPGR diet (*p* = 0.05; Table 2). Fecal output expressed on a DM basis was greater for cats fed the MediumPGR diet than cats fed the HighPGR diet (*p* = 0.02, Table 2), and both diets did not differ from the LowPGR diet. Urinary N was greater (*p* < 0.01, Table 2) for the LowPGR diet than the Medium and the HighPGR diet. Fecal output was a function of feed intake, and therefore when output was normalized based on intake, differences no longer existed indicating that the more a cat consumed, the more fecal matter was produced. Daily food intake was greater for cats receiving the Medium and LowPGR diets (*p* < 0.001) than the HighPGR diet (Table 3), but this was due to differences in ME values among diets. On a daily basis, GE intake was greatest (*p* < 0.001; Table 3) for cats fed the LowPGR diet, intermediate for cats fed the MediumPGR diet and lowest for the HighPGR diet.

Dry matter digestibility was similar among diets, whereas organic matter digestibility was greater (*p* = 0.03, Table 3) for cats fed the HighPGR diet than cats fed the MediumPGR diet, and neither treatment differed from cats fed the LowPGR diet. Protein digestibility was greatest (*p* < 0.01, Table 3) in cats fed the LowPGR diet, followed by cats fed the HighPGR diet and was lowest for cats fed the MediumPGR diet. Fat digestibility was greater (*p* < 0.01, Table 3) for cats fed the Low and MediumPGR diets than cats fed the HighPGR diet. Dry matter disappearance (DMD) was greatest for cats fed the LowPGR diet, followed by cats fed the HighPGR diet, and then cats fed the MediumPGR diet. Since DMD was determined using in vitro digestibility, only one measurement was recorded, and we can thus not comment on statistical significance of these differences; however, the DMD digestibility values were 92.7, 91.1, and 90.7% for the Low, High and MediumPGR diets, respectively, and were similar to in vivo measurements of DM digestibility (Table 3). On average, in vitro dry matter disappearance overestimated apparent dry matter digestibility by 5.0%.

Per unit of feed, the GE, urinary energy, and measured ME value were greatest (*p* < 0.001; Table 4) for cats fed the LowPGR diet. The GE and measured ME value were greatest for the LowPGR diet (*p* < 0.001), followed by the MediumPGR diet, and lowest for the HighPGR diet. The UE was greatest for cats fed the LowPGR diet (*p* < 0.001), with intermediate values for cats fed the HighPGR diet, and lowest for cats fed the MediumPGR diet.

Measured ME is compared with ME calculated using both the traditional and modified Atwater equations (Table 5). The measured ME value was greatest (*p* < 0.05; Table 5) for the LowPGR diet, intermediate for the MediumPGR diet, and lowest for the HighPGR diet. The ME value calculated using both ME equations followed the same ranking as the measured ME value. Because only one number per diet can be calculated, we could not assess this numerical ranking statistically. The measured ME values were greater than calculated ME values for all 3 diets (Table 5). Thus, both modified and traditional Atwater equations underestimate ME. The modified Atwater equation underestimated measured ME values by approximately 12% for all diets (11.9, 10.8, and 13.6% for High, Medium and LowPGR, respectively). The traditional Atwater equation had the closest prediction to the measured ME values (2.0, 1.5, and 4.6% below measured ME for High, Medium and LowPGR, respectively) (Table 5). Though not included in our hypotheses, ME was also calculated using the NRC equation that accounts for crude fiber, and this method underestimated by 8% on average (11.3, 6.0, and 7.8% below measured ME for High, Medium and LowPGR, respectively).

## 4. Discussion

This study was the first to show similarity among in vivo and in vitro measures of NFE, and that high quality diets have similar digestibility regardless of ingredient selection. Similar to previous literature, the modified Atwater equation does not accurately predict measured ME content of the cat diets investigated in the present study.

The observed differences in intake among diets were expected. Aforementioned, cats were fed once daily to 95% of their individual maintenance requirements. Although this is not common for cats outside of a research setting, this was done to encourage consumption and ensure that differences in intake were due to diet alone. We offered food isocalorically and intake differences were due to food refusals that likely occurred when cats reached a level of carbohydrate intake close to their “carbohydrate ceiling” [16]. This ceiling is a level of carbohydrate intake beyond which food intake is reduced, suggesting that cats may only be able to metabolize ingested carbohydrates up to a definitive level. This ceiling occurs at approximately 300 kJ/day [16]; however, the ceiling of intake was roughly 244 kJ/day in the present study. Indeed, other variances in individual cats, experimental methods, or the environment may have contributed to the differences among studies. Other studies indicated that when provided ad libitum access to feed, cats fed high fat or high protein diets, rather than high carbohydrate diets, are more prone to weight gain [17,18]. These studies support the existence of a “carbohydrate ceiling”, resulting in marginal intakes of high carbohydrate diets compared with diets high in fat or protein [16]. Notably, neutral detergent fiber (NDF) and crude fiber (CF) did not impact food consumption in the present study, indicating that intake was affected largely by differences in total NFE.

Differences in protein digestibility in the present study are related to dietary protein intake and similar to other species. For example, apparent crude protein digestibility, nitrogen (N) intake, and N retention increased with increasing dietary protein level in mink [19]. Similarly, horses consuming 865 g/day of crude protein increased their CP digestibility 5% compared to horses consuming 840 g/day [20]. However, calculating crude protein measurements and apparent digestibility values has various limitations that need to be considered.

Crude protein calculations assume that all nitrogen is protein bound, and do not account for sources of non-protein nitrogen. Indeed, crude protein values may overestimate net (true) protein content by up to 20.6% in certain foods [21]. Additionally, a potential drawback of determining apparent crude protein digestibility is the limited ability to comment on true protein digestibility. Apparent crude protein digestibility does not account for endogenous amino acid losses at the terminal ileum [22,23]. The presence of anti-nutritional factors such as protease inhibitors, lectins, saponins, and phytate may increase the endogenous losses of dietary amino acids in pigs and humans [24,25,26], and this presumably extends to cats. Only true ileal digestibility measurements can accurately account for endogenous losses and are thus recognized to more accurately define dietary content of amino acids to support bodily functions than total tract digestibility values [23]. However, ileal digesta measurements are invasive and difficult to manage; thus, feces were used to estimate apparent total tract digestibility in the present study.

Studies in pigs have indicated that the apparent digestibility of fat increases with increasing amounts of fat in the diet [27]. This increase supports results in the present study, as fat digestibility is augmented by increasing dietary fat content. This may also explain the lack of difference in fat digestibility between the Medium and LowPGR diets, as the two diets were similar in crude fat content. Furthermore, lipid type and processing conditions will affect apparent fat digestibility. In rats, liquid oils are absorbed more readily than solid triacylglycerols [28]. Notably, lipid type may have contributed to fat digestibility differences observed in the present study. While all diets contained fat from animal sources such as fat preserved with mixed tocopherols, the Medium and LowPGR diets contained major fat contributions from fish oil and sunflower oil, respectively. Non-hydrogenated palm oil was retained at 99.6% in rats [28] indicating that the inclusion of non-hydrogenated oils in the Medium and LowPGR diets may have contributed to the greater fat digestibility values in these diets than the HighPGR diet.

Apparent OM digestibility measurements approximated carbohydrate digestibility in the present study. Previously, apparent OM digestibility correlated negatively to fiber content of dog and cat foods [29]. The addition of 40% apple pomace, a high fiber ingredient, to a meat-based diet with 0% analyzed crude fiber decreased OM digestibility from 85.5 to 56.9% in cats [30]. However, crude fiber content did not seem to dictate apparent OM digestibility in the present study, indicating that our results may not be biologically relevant, or that all diets were below a physiological maximum for fiber. Regardless, these OM digestibly measurements indicate that, despite the inherent obligatory carnivorous characteristics of domestic cats, they can efficiently digest diets with significant contributions from carbohydrates.

Our digestibility values are similar to those reported in literature. Ahlstrom and Skrede [31] conducted a nutrient digestibility experiment in dogs, using multiple diets ranging from 20.6 to 43.0% in starch. Overall, the mean apparent digestibility of dry matter, protein and fat among diets was 85.5, 84.8, and 95.8%, respectively. In both intact and neutered male cats fed a high carbohydrate diet (27% starch), dry matter, organic matter, crude protein, and crude fat corresponded to digestibility of 84.5, 86.0, 82.0, and 95.3%, respectively [32]. Furthermore, apparent starch digestibility values were >93% in cats fed 6 different diets containing 35% starch [2]. The results of these studies agree with the present study demonstrating that starch can be digested readily by cats, and does not reduce digestibility of other macronutrients.

Dry matter disappearance was also measured in vitro to allow us to compare to our in vivo measurements (Table 3). The in vivo and in vitro digestibility measurements were numerically similar, indicating that in vitro digestion can be used to predict in vivo digestibility. This may allow for rapid screening of novel ingredients or processing methods prior to placing an in vivo study.

The results of the fecal and urine analyses varied minimally among diets. Previous research indicates an increase in urinary nitrogen excretion when nitrogen intakes are increased beyond theoretical requirements [33,34]. In the present study, daily urinary energy excretion did not differ, but cats fed the LowPGR diet had the greatest urinary N excretion. Cats consuming LowPGR had the greatest protein intake, resulting in the greatest urinary energy loss per kg of diet. Our study diets exceeded the recommended protein contribution for adult cats, and the measurements of urinary nitrogen indicate that protein was supplied in excess of the cats’ protein requirements from at minimum, the LowPGR diet [7]. Additionally, fecal score and fecal dry matter proportion did not differ among treatments indicating that our cats had a solid function and health of the gastrointestinal tract, nutrient absorption, and a consistent colonic environment. For all three diets, fecal scores of cats were within the ideal score [35] indicating that cats were able to digest/ferment these diets, regardless of NFE level.

In the present study, ME values calculated using both Atwater and modified Atwater equations underestimated measured ME values among all diets. The discrepancy mimics research, and indicates that the modified Atwater calculation is inappropriate for calculating a ME value to determine daily food allowance for cats. In dogs, the modified Atwater equation underestimated the ME value of low-ash poultry meal by 15% [36]. Furthermore, predicted ME values underestimated in vivo values of dog food with ME values above 3.6 kcal ME/g DM, which corresponds to most dry extruded pet foods currently on the market [37,38,39]. Conversely, the modified Atwater equation has accurately predicted ME value in some studies. Digestibility studies of commercial and non-commercial pet foods predicted ME values with an error of 0.16% for dogs and 1.57% for cats [40]. However, those studies included both wet and dry foods of varying quality, with lower dry matter, fat, carbohydrate, and energy digestibility compared with the present study diets. Additionally, Hall et al. [40] calculated ME using a correction factor for energy lost in urine (0.86 × g protein absorbed). This correction may explain why calculated energy density was more accurate than predicted values in the present study.

While the modified Atwater equation may accurately predict energy values for less digestible, average quality pet foods, its use for premium or super-premium diets may underestimate true energy values of feed to calculate feeding requirements. This discrepancy is a prevalent issue in today’s society, as the market share of premium and super-premium pet foods is growing disproportionately to other segments of products that are lower in price and purported quality [41]. Moreover, pet food has generally improved in quality and digestibility since the Atwater equations were first developed. Thus, using these current standards to calculate ME values of diets may cause overfeeding and subsequent weight gain of pets due to a caloric surplus.

Because cat foods are generally more energy dense than dog foods, we recommend instead using the traditional Atwater to calculate ME for all cat diets. Alternative methods may also be considered to predict diet ME values more accurately, such as our in vitro DMD assay or the NRC ME prediction equations. Notably, a recent Canadian labeling policy has been approved that requires pet food manufacturers to report total dietary fiber [42]. With the adoption of this policy, standards may begin to shift towards using the NRC predictive ME equations that include total dietary fiber instead of the Atwater models. Regardless, various calculations and theories have been proposed as better predictors of diet ME values than the modified or traditional Atwater equations, but one has yet to become the new standard for regulatory guidance.

In conclusion, the present study supported previous research that cats can efficiently digest diets with major contributions from carbohydrates. Despite differences in digestibility among diets, all diets were highly digestible. Data from in vitro and in vivo digestibility methods demonstrated that in vitro techniques can potentially be used in place of in vivo studies to rapidly screen novel ingredients or processing methods. Furthermore, we confirmed that the modified Atwater equation does not give an accurate estimate of ME of high quality pet foods and may not be appropriate for any cat diet due to the overall higher energy density compared to dog foods. We recommended that the traditional Atwater coefficients are used to calculate diet ME values to avoid errors in feeding guidelines and subsequent weight gain in pets until future research establishes a more accurate equation to calculate the ME value of cat food.

## Figures and Tables

**Table 1:** Analyzed nutrient composition of the 3 experimental diets differing in perceived glycemic response (PGR) ^1^.

Item	HighPGR ^2^	MediumPGR ^3^	LowPGR ^4^
Moisture, %	7.16	6.76	5.31
Ash, %	6.36	6.31	6.38
Crude protein ^5^, %	38.02	35.86	42.06
Crude fat, %	10.83	20.02	20.42
Nitrogen-free extract ^6^, %	34.1	29.5	23.6
Starch ^7^, %	36.75	30.72	23.56
Crude fiber, %	1.17	1.78	2.58
Acid detergent fiber, %	1.88	2.95	2.43
Neutral detergent fiber, %	7.36	12.58	10.57
Available lysine, %	1.62	1.91	2.80
GE, kcal/kg	4916	5253	5462
Calculated ME ^8^	3752	4081	4137

^1^ Each diet was analyzed in triplicate. Results (except moisture) presented on a dry-matter basis. ^2^ HighPGR was Purina ONE Chicken and Rice (Nestlé, St. Louis, MO, USA) containing as main ingredients: chicken, brewer’s rice, corn gluten meal, poultry by-product meal, wheat flour, animal fat preserved with mixed-tocopherols, whole grain corn, soy protein isolate, fish meal, animal liver flavor, KCl, H_3_PO_4_, CaCO_3_, caramel color, choline chloride, and salt. ^3^ MediumPGR was Iams Kitten Proactive Health (Procter & Gamble, Cincinatti, OH) containing as main ingredients: chicken, chicken by-product meal, corn meal, chicken fat preserved with mixed tocopherols, dried beet pulp, ground whole grain sorghum, dried egg product, natural flavor, fish oil preserved with mixed tocopherols, KCl, fructooligosaccharides, choline chloride, CaCO_3_, brewer’s dried yeast, DL-Met, and salt. ^4^ LowPGR was Innova (Procter & Gamble, Cincinatti, OH) containing as main ingredients: turkey, chicken, chicken meal, whole grain barley and whole grain brown rice, chicken fat preserved with mixed tocopherols, peas, natural flavors, apples, herring, flaxseed, eggs, blueberries, pumpkin, tomatoes, sunflower oil, KCl, DL-Met, carrots, pears, cranberries, menhaden oil, cottage cheese, taurine, green beans, alfalfa sprouts, parsnips, and salt. ^5^ Percentage N × 6.25. ^6^ NFE (%) = 100 − moisture (%) − protein (%) − fat (%) − fiber (%) − ash (%). ^7^ Determined using AOAC Official Method 979.10. ^8^ Calculated with modified Atwater equation (AAFCO, 1997): ME (kcal/kg) = 10 × (3.5 × Crude Protein % + 8.5 × Crude Fat % + 3.5 × Nitrogen-Free Extract %).

**Table 2:** Feces and urine characteristics of cats fed 3 experimental diets differing in perceived glycemic response (PGR).

Item	HighPGR	MediumPGR	LowPGR	SEM ^1^	*p*-Value
Fecal score ^2^	4.0	4.0	3.7	0.06	0.229
Fecal output (wet), g/day	12.2 ^a^	15.5 ^b^	16.1 ^c^	3.95	0.050
Fecal DM, %	39.7	37.3	36.8	1.03	0.328
Fecal output (100% DM), g/day	4.8 ^a^	5.8 ^b^	5.5 ^a,b^	0.20	0.022
Fecal output (wet), g/day	0.07	0.08	0.08	0.01	0.369
Fecal output (wet) (g)/100 g DM intake	30.8	36.7	37.3	1.65	0.267
Fecal energy (cal/g day^−1^)	3576	3555	3478	24.7	0.243
Urine energy (cal/g day^−1^)	155	143	172	9.16	0.437
Urine N (mg/mL)	3.52 ^a^	3.36 ^a^	4.12 ^b^	0.10	0.003

^a–c^ Within a row, means without a common superscript differ (*p* < 0.05). ^1^ SEM: Standard error of the mean. Means were based on 12 cat observations per diet. ^2^ Scores based on the following scale: 1 = watery, liquid that can be poured; 2 = unformed stool; 3 = soft, moist, formed stool; 4 = dry, well-formed stool; 5 = dry, hard pellets.

**Table 3:** Intake and total tract digestibility of nutrients in cats of three experimental diets differing in perceived glycemic response (PGR).

Item	HighPGR	MediumPGR	LowPGR	SEM ^1^	*p*-Value
Food intake, g/day	42.8 ^a^	45.0 ^b^	45.1 ^b^	1.2	<0.001
Protein intake, g/day	14.9 ^a^	15.0 ^a^	17.6 ^b^	0.49	<0.001
Fat intake, g/day	6.7 ^a^	10.0 ^b^	10.3 ^c^	0.37	<0.001
Fiber intake, g/day	0.8 ^a^	1.1 ^b^	1.4 ^c^	0.05	<0.001
NFE ^2^ intake, g/day Calculated NFE intake (kJ/day) ^3^	14.6 ^a^ 244.4	13.3 ^b^ 222.6	10.6 ^c^ 177.4	0.45	<0.001
GE intake, kcal/day	210.2 ^a^	236.4 ^b^	246.2 ^c^	6.90	<0.001
DM digestibility, %	87.6	86.2	87.0	0.41	0.128
DMD ^4^, %	91.14	90.74	92.70		
OM digestibility, %	90.9 ^a^	89.5 ^b^	90.3 ^a,b^	1.03	0.031
Protein digestibility, %	88.7 ^a^	87.3 ^b^	91.4 ^c^	0.49	<0.001
Fat digestibility, %	92.9 ^a^	95.4 ^b^	95.0 ^b^	0.25	<0.001

^a–c^ Within a row, means without a common superscript differ (*p* < 0.05). ^1^ SEM: Standard error of the mean. Means were based on 12 cat observations per diet. ^2^ NFE = N-free extract. ^3^ Caloric value calculated by multiplying NFE intake by 4 kcal, noting that 1 g of carbohydrate provides 4 kcal of energy [11]. ^4^ Dry matter disappearance. Determined using in vitro simulated digestion procedure [15].

**Table 4:** Intake and excretion (as is basis and per 100 g) of energy of cats of 3 experimental diets differing in perceived glycemic response (PGR).

Name	HighPGR	MediumPGR	LowPGR	SEM ^1^	*p*-Value
Fecal energy, kcal/day	18.97	23.12	21.37	0.82	0.116
Urinary energy, kcal/day	11.42 ^a^	11.25 ^a^	13.86 ^b^	0.41	0.011
GE, kcal/100 g diet	491.6 ^a^	525.3 ^b^	546.9 ^c^	3.2	<0.001
Fecal energy, kcal/100 g diet	44.37	50.38	47.46	1.6	0.353
Urinary energy, kcal/100 g diet	19.84 ^a^	17.31 ^b^	20.02 ^c^	0.4	<0.001
Measured ME, kcal/100 g diet	426.0 ^a^	457.4 ^b^	478.7 ^c^	3.6	<0.001
Calculated ME ^2^, kcal/100 g diet	375.2	408.1	413.7		

^a–c^ Within a row, means without a common superscript differ (*p* < 0.05). ^1^ Means were based on 12 cat observations per diet. ^2^ Calculated with modified Atwater equation (AAFCO, 1997): ME (kcal/kg) = 10 × (3.5 × Crude Protein % + 8.5 × Crude Fat % + 3.5 × Nitrogen-Free Extract %).

**Table 5:** Comparison of measured ME with ME calculated using Atwater and modified Atwater equations, and resulting caloric surplus per day for 3 experimental diets differing in perceived glycemic response (PGR) fed to cats.

Name	HighPGR	MediumPGR	LowPGR
Measured ME, kcal/kg as fed	4259 ^a^	4574 ^b^	4787 ^c^
Calculated ME, kcal/kg as fed			
Modified Atwater ^1^	3752	4081	4137
Traditional Atwater ^2^ NRC ^3^	4176 3778	4505 4301	4565 4413
**Calorie surplus per day (kcal/day)**			
Modified Atwater	21.7	22.2	29.3
Traditional Atwater NRC	3.6 21.1	3.1 12.1	10 16.4
**Calorie surplus per day, %**			
Modified Atwater	11.9	10.8	13.6
Traditional Atwater NRC	2.0 11.3	1.5 6.0	4.6 7.8

^a–c^ Within a row, means without a common superscript differ (*p* < 0.05). ^1^ Calculated with modified Atwater equation (AAFCO, 1997): ME (kcal/kg) = 10 × (3.5 × Crude Protein % + 8.5 × Crude Fat % + 3.5 × Nitrogen-Free Extract %). ^2^ Calculated with traditional Atwater equation (Atwater, 1902): ME (kcal/kg) = 10 × (4 × Crude Protein % + 9 × Crude Fat % + 4 × Nitrogen-Free Extract %). ^3^ Calculated using the NRC predictive equations (NRC, 2006): Step 1: GE (kcal) = (5.7 × g protein) + (9.4 × g fat) + 4.1 × (g NFE + g fiber); Step 2: Percentage energy digestibility = 87.9 − (0.88 × percentage crude fiber in dry matter); Step 3: DE (kcal/g) = (GE × percentage energy digestibility/100); Step 4: ME (kcal/g) = DE − (0.77 × g protein).

## References

[1] Verbrugghe A., Hesta M., Daminet S., Polis I., Holst J.J., Buyse J., Wuyts B., Janssens G.P.J. (2012). Propionate absorbed from the colon acts as gluconeogenic substrate in a strict carnivore, the domestic cat (felis catus). J. Anim. Physiol. Anim. Nutr..

[2] De-Oliveira L.D., Carciofi A.C., Oliveira M.C.C., Vasconcellos R.S., Bazolli R.S., Pereira G.T., Prada F. (2008). Effects of six carbohydrate sources on diet digestibility and postprandial glucose and insulin responses in cats. J. Anim. Sci..

[3] Gooding M.A., Atkinson J.L., Duncan I.J.H., Niel L., Shoveller A.K. (2015). Dietary fat and carbohydrate have different effects on body weight, energy expenditure, glucose homeostasis and behaviour in adult cats fed to energy requirement. J. Nutr. Sci..

[4] Forrester S., Kirk C. Cats and carbohydrates—What are the concerns?. Proceedings of the NAVC Conference.

[5] Tran Q.D., Hendriks W.H., van der Poell A.F.B. (2008). Effects of extrusion processing on nutrients in dry pet food. J. Sci. Food Agric..

[6] Livesey G. (2001). A perspective on food energy standards for nutrition labelling. Br. J. Nutr..

[7] AAFCO (2012). Association of American Feed Control Officials: Official Publication.

[8] Atwater W.O. (1916). Principles of Nutrition and Nutritive Value of Foods.

[9] AAFCO (1997). Association of American Feed Control Officials: Official Publication.

[10] Kendall P.T., Holme D.W., Smith P.M. (1982). Comparative-evaluation of net digestive and absorptive efficiency in dogs and cats fed a variety of contrasting diet types. J. Small Anim. Pract..

[11] NRC (2006). Nutrient Requirements of Dogs and Cats.

[12] Rand J., Farrow H., Fleeman L., Appleton D. Diet in the Prevention of Diabetes and Obesity in Companion Animals. http://apjcn.nhri.org.tw/server/apjcn/procnutsoc/2000+/2003/Rand.pdf.

[13] Miller J.B., Pang E., Bramall L. (1992). Rice—A high or low glycemic index food. Am. J. Clin. Nutr..

[14] AOAC (1997). Official Methods of Analyses.

[15] Huang G., Sauer W.C., He J., Hwangbo J., Wang X. (2003). The nutritive value of hulled and hulless barley for growing pigs. 1. Determination of energy and protein digestibility with the in vivo and in vitro method. J. Anim. Feed Sci..

[16] Hewson-Hughes A.K., Hewson-Hughes V.L., Miller A.T., Hall S.R., Simpson S.J., Raubenheimer D. (2011). Geometric analysis of macronutrient selection in the adult domestic cat, felis catus. J. Exp. Biol..

[17] Backus R.C., Cave N.J., Keisler D.H. (2007). Gonadectomy and high dietary fat but not high dietary carbohydrate induce gains in body weight and fat of domestic cats. Br. J. Nutr..

[18] Coradini M., Rand J.S., Morton J.M., Rawlings J.M. (2011). Effects of two commercially available feline diets on glucose and insulin concentrations, insulin sensitivity and energetic efficiency of weight gain. Br. J. Nutr..

[19] Jiang Q., Li G., Zhang T., Zhang H., Gao X., Xing X., Zhao J., Yang F. (2015). Effects of dietary protein level on nutrients digestibility and reproductive performance of female mink (neovison vison) during gestation. Anim. Nutr..

[20] Graham-Thiers P.M., Bowen L.K. (2011). Effect of protein source on nitrogen balance and plasma amino acids in exercising horses. J. Anim. Sci..

[21] SaloVaananen P.P., Koivistoinen P.E. (1996). Determination of protein in foods: Comparison of net protein and crude protein (nx6.25) values. Food Chem..

[22] Bryden W.L., Li X. Amino Acid Digestibility and Poultry Feed Formulation: Expression, Limitations and Application. http://www.scielo.br/pdf/rbz/v39sspe/31.pdf.

[23] Schaafsma G. (2000). The protein digestibility-corrected amino acid score. J. Nutr..

[24] Caine W.R., Sauer W.C., Tamminga S., Verstegen M.W.A., Schulze H. (1997). Apparent ileal digestibilities of amino acids in newly weaned pigs fed diets with protease-treated soybean meal. J. Anim. Sci..

[25] Rowan A.M., Moughan P.J., Wilson M.N., Maher K., Tasmanjones C. (1994). Comparison of the ileal and fecal digestibility of dietary amino-acids in adult humans and evaluation of the pig as a model animal for digestion studies in man. Br. J. Nutr..

[26] Hughes R.J., Choct M. (1999). Chemical and physical characteristics of grains related to variability in energy and amino acid availability in poultry. Aust. J. Agric. Res..

[27] Kane E., Morris J.G., Rogers Q.R. (1981). Acceptability and digestibility by adult cats of diets made with various sources and levels of fat. J. Anim. Sci..

[28] Wang Y., Corwin R., Jaramillo D.P., Wojnicki F.J., Coupland J.N. (2011). Acceptability and digestibility of emulsions in a rat model: Effects of solid fat content and lipid type. J. Am. Oil Chem. Soc..

[29] Earle K.E., Kienzle E., Opitz B., Smith P.M., Maskell I.E. (1998). Fiber affects digestibility of organic matter and energy in pet foods. J. Nutr..

[30] Fekete S., Hullar I., Andrasofszky E., Rigo Z., Berkenyi T. (2001). Reduction of the energy density of cat foods by increasing their fibre content with a view to nutrients' digestibility. J. Anim. Physiol. Anim. Nutr..

[31] Ahlstrom O., Skrede A. (1998). Comparative nutrient digestibility in dogs, blue foxes, mink and rats. J. Nutr..

[32] Thiess S., Becskei C., Tomsa K., Lutz T.A., Wanner M. (2004). Effects of high carbohydrate and high fat diet on plasma metabolite levels and on iv glucose tolerance test in intact and neutered mate cats. J. Feline Med. Surg..

[33] Marini J.C., Van Amburgh M.E. (2003). Nitrogen metabolism and recycling in holstein heifers. J. Anim. Sci..

[34] Tome D., Bos C. (2000). Dietary protein and nitrogen utilization. J. Nutr..

[35] Kerr K.R., Boler B.M.V., Morris C.L., Liu K.J., Swanson K.S. (2012). Apparent total tract energy and macronutrient digestibility and fecal fermentative end-product concentrations of domestic cats fed extruded, raw beef-based, and cooked beef-based diets. J. Anim. Sci..

[36] Yamka R.M., McLeod K.R., Harmon D.L., Freetly H.C., Schoenherr W.D. (2007). The impact of dietary protein source on observed and predicted metabolizable energy of dry extruded dog foods. J. Anim. Sci..

[37] Castrillo C., Hervera M., Dolores Baucells M. Methods for Predicting the Energy Value of Pet Foods. http://www.scielo.br/scielo.php?pid=S1516-35982009001300001&script=sci_arttext&tlng=es.

[38] Kienzle E., Opitz B., Earle K.E., Smith P.M., Maskell I.E., Iben C. (1998). The development of an improved method of predicting the energy content in prepared dog and cat food. J. Anim. Physiol. Anim. Nutr..

[39] Laflamme D.P. (2001). Determining metabolizable energy content in commercial pet foods. J. Anim. Physiol. Anim. Nutr..

[40] Hall J.A., Melendez L.D., Jewell D.E. (2013). Using gross energy improves metabolizable energy predictive equations for pet foods whereas undigested protein and fiber content predict stool quality. PLoS ONE.

[41] (2017). Pet Care in Canada.

[42] Bureau of Nutritional Sciences (2017). Policy for Labelling and Advertising of Dietary Fiber-Containing Food Products.

